# G-Quadruplex Linked DNA Guides Selective Transfection into Nucleolin-Overexpressing Cancer Cells

**DOI:** 10.3390/pharmaceutics14102247

**Published:** 2022-10-21

**Authors:** Mengxi Xiang, Yongkui Li, Jia Liu, Jie Shi, Yizhi Ge, Chen Peng, Yawen Bin, Zheng Wang, Lin Wang

**Affiliations:** 1 Research Center for Tissue Engineering and Regenerative Medicine, Union Hospital, Tongji Medical College, Huazhong University of Science and Technology, Wuhan 430022, China; 2 Department of Gastrointestinal Surgery, Union Hospital, Tongji Medical College, Huazhong University of Science and Technology, Wuhan 430022, China; 3 Department of Clinical Laboratory, Union Hospital, Tongji Medical College, Huazhong University of Science and Technology, Wuhan 430022, China

**Keywords:** gene delivery, cancer cell selectivity, transfection, nucleolin

## Abstract

Gene therapy is a promising approach for treating tumors. Conventional approaches of DNA delivery depending on non-viral or viral vectors are unsatisfactory due to the concerns of biosafety and cell-targeting efficiency. The question how to deliver DNA into tumor cells efficiently and selectively is a major technological problem in tumor gene therapy. Here, we develop a vector-free gene transfer strategy to deliver genes effectively and selectively by taking advantage of targeting nucleolin. Nucleolin, a shuttle protein moving between cell membrane, cytoplasm and nuclei, is overexpressed in tumor cells. It has a natural ligand G-quadruplex (Gq). Gq-linked DNA (Gq-DNA) is likely to be internalized by ligand dependent uptake mechanisms independently of vectors after neutralizing negative charges of cell membrane by targeting nucleolin. This strategy is referred to as Gq-DNA transfection. Benefiting from its high affinity to nucleolin, Gq-DNA can be effectively delivered into nucleolin-positive tumor cells even nuclei. Gq-DNA transfection is characterized by low cytotoxicity, high efficiency, ease of synthesis, high stability in serum, direct access into nuclei, and specific nucleolin-positive tumor cell targeting.

## 1. Introduction

One of the hallmarks of cancer is genome mutation [1]. To target genetic changes of tumorigenesis, exogenetic nucleic acids are delivered into tumor cells to rescue or correct genetic changes, which is defined as tumor gene therapy [2]. A key challenge of tumor gene therapy is how to achieve DNA delivery safely, efficiently and selectively. Conventional approaches of DNA delivery include virus-mediated methods and non-viral methods. Virus-mediated methods are efficient, but its application is limited because of the concerns regarding biosafety, including immunogenicity and risk of randomly insertional mutagenesis [3,4,5]. Non-viral methods usually rely on physical stimulations and non-viral vectors. Physical stimulations mainly refer to electroporation, which is efficient but only applicable in vitro and sonoporation, which is noninvasive but less effective than electroporation [6]. The main drawbacks of non-viral vectors (including lipids, polymers, peptides and inorganic nanoparticles) are poor selectivity and low delivery efficacy [7,8]. One of the common reasons for low delivery efficacy is the barrier of nuclear entry. Exo-genetic nucleic acids are mostly delivered into cytoplasm through cell membrane, not directly into nuclei, which means that the expression of the delivered genes depends on cell proliferation and mitosis [9,10].

Nucleolin, a shuttle protein moving between cell membrane, cytoplasm and nuclei, is overexpressed on cell membrane in many kinds of cancer cells [11,12]. Thus, we assumed that nucleolin-targeting gene delivery system could effectively target tumor cells and deliver gene drugs directly into nuclei, which could enhance tumor selectivity and delivery efficacy at the same time. The special DNA structure G-quadruplex (Gq) could bind to nucleolin with high affinity [13,14]. Gq structure is formed in G-rich region in vivo through stacked guanine tetrads (G-quartets) which consists of Hoogsteen hydrogen bonding by every four guanines [15]. Unique 3D structure of Gq makes it resistant to nuclease degradation and keep it stable in serum [16,17]. Therefore, Gq can be used to construct tumor selective gene delivery system because of its stability in serum and high affinity binding with nucleolin [18,19].

Herein, we developed a low cytotoxic, effective and selective vector-free gene transfer strategy by directly linking Gq to DNA (Gq-DNA). The Gq-linked enhanced green fluorescent protein (EGFP) and luciferase (Luc) genes were specifically taken in by nucleolin-positive tumor cells, and exhibited higher transfection efficacy than commercial transfection reagents, such as polyethyleneimine (PEI) and Invitrogen Lipofectamine 2000 (Lipo2000). Importantly, Gq-DNA transfection showed higher transfection efficiency in hepatoma cell line HepG2 than normal hepatocytes L02, demonstrating its tumor targeting capability. Thus, Gq-DNA transfection provided a new transfection method for nucleolin-overexpressing cancer cells.

## 2. Materials and Methods

### 2.1. DNA Linearization

To prepare Gq-linked DNA, linear DNA fragments were amplified by conventional Polymerase Chain Reaction (PCR) from plasmid pEGFP-C1 or pCMV-tag2B-luciferase with the primer TTTTGCTCACATGTTCTTTC (forward) and ATTTACGCGTTAAGATA (reverse). The PCR procedure was carried out by Phanta^®^ Max Super-Fidelity DNA Polymerase (Vazyme Biotech, Nanjing, China). The PCR products were analyzed on 1.5% agarose gel and recovered with a Gel Extraction Purification Kit (Omega, Norwalk, CT, USA).

### 2.2. Amplification of Single Strand DNA

Taking the linear DNA fragment as the template, the single strand DNA was amplified with the asymmetric PCR which only used forward or reverse primer separately. The asymmetric PCR reaction system was as follows: 25 μL of 2 × Phanta Max Buffer, 1 μL of dNTP Mix (10 mM each), forward primer (10 μM) or reverse primer (10 μM) 2 μL, template (100~200 ng), Phanta Max Super-Fidelity DNA Polymerase 1 μL and adding ddH2O up to 50 μL. The PCR procedure was as follows: 95 °C for 5 min; 35 cycles of 95 °C for 30 s, 50.6 °C for 30 s and 72 °C 105 s, 72 °C 5 min. The primer sequences for asymmetric PCR were GGTGGTGGTGGTTGTGGTGGTGGTGGTTTTTTTTTTTTTGCTCACATGTTCTTTC (forward) and GGTGGTGGTGGTTGTGGTGGTGGTGGTTTTTTTTTATTTACGCGTTAAGATA (reverse).

### 2.3. Generation and Purification of Gq-DNA

The two products of each single strand were mixed by the ratio of 1:1 per 100 μL mixture of the single strand products and 4 μL AS1411 (100 μM) strands were dissolved in 25 μL 5× annealing buffer (140 mM Tris-HCl, 1 M KCl, and 20 mM MgCl_2_) [20,21,22]. The sequence of AS1411 is GGTGGTGGTGGTTGTGGTGGTGGTGG. The mixture was annealed from 95 °C to 25 °C with a 3 min pause at every 5 °C reduction. The product was purified by DNA electrophoresis with a PCR Product Purification kit (SIMGEN, Hangzhou, China).

### 2.4. Cells and Cell Culture

Human hepatoma cell line HepG2, human colon cancer cell line HCT116, human breast cancer cell line MCF7 and mouse melanoma cell line B16F10 were obtained from ATCC. Human normal liver cell line L02 was obtained from CCTCC. The normal immortalized colonic epithelial cell line NCM460 was obtained from INCELL. All these cells were incubated in DMEM (HyClone, Logan, UT, USA) containing 10% fetal bovine serum, 100 IU/mL penicillin and 100 μg/mL streptomycin at 37 °C in incubator with 5% CO_2_.

### 2.5. Serum Stability Assay

Gq-DNA or DNA were incubated in DMEM containing 5% fetal bovine serum. After incubation for 12, 24, 36 and 48 h at 37 °C, the samples were analyzed by DNA electrophoresis in a 1.5% agarose gel and stained with ethidium bromide (EB).

### 2.6. DNA Delivery

The conventional transfection with commercial reagent Lipofectamine 2000 (Thermo Fisher, Waltham, MA, USA) followed the provided instructions. For transfection with polyethyleneimine (PEI, Polysciences, Warrington, PA, USA), 5 × 10^4^ cells were seeded on 24-well plates and grew to 70% confluence. The medium was changed with serum-free DMEM half an hour before transfection. Then, 0.8 μg DNA and 3 μL PEI were sufficiently mixed into 50 μL serum-free DMEM in tube, and incubated at room temperature for 20 min. Then, the mixture was dripped into cell culture well slowly. Six hours later, the medium was changed into DMEM containing 10% fetal bovine serum.

For Gq-DNA transfection, cells were seeded on 24-well plates and grew to 70% confluence. The cells cultured in DMEM containing 10% fetal bovine serum or serum free DMEM were incubated with polybrene (5 μg/mL) and 0.8 μg Gq-DNA (or DNA as control) for 6 h. Then, the medium was changed into fresh DMEM containing 10% fetal bovine serum.

### 2.7. Fluorescent Staining

HepG2 or B16F10 cells were transfected with DNA to express GFP or DNA stained with YOYO-1 (Thermo Fisher Scientific, Waltham, MA, USA). The samples were harvested, fixed with 4% paraformaldehyde and stained with DAPI (4′, 6-diamidino-2-phenylindole) for 15 min. The fluorescent images were obtained by Nikon Ti-U microscope equipped with a CSU-X1 spinning-disk confocal unit (Yokogawa, Tokyo, Japan) and an EM-CCD camera (iXon+; Andor, Belfast, Northern Ireland).

### 2.8. Flow Cytometry

The samples were harvested and washed with FACS buffer containing 5% FBS for 3 times and fixed with 2% paraformaldehyde diluted with FACS buffer containing 5% FBS. The results were analyzed by Flow cytometry using BD LSRFortessa X-20 flow cytometer.

### 2.9. Luciferase Assay

HepG2 or B16F10 cells were seeded on 24 well plates and transfected with DNA to express luciferase. Then, 24 h later, cells were lysed with 100 μL lysis buffer on the ice for 30 min. The mixture was centrifuged for 5 min at 12,000 rpm in 4 °C. Additionally, the supernatant was collected to detect the luciferase activities. Luciferase activities were normalized with protein concentrations.

### 2.10. Cellular Uptake

To test cellular uptake efficiency of the traditional transfection method and Gq-DNA transfection, DNA and Gq-DNA stained with YOYO-1 (Thermo Fisher Scientific, Waltham, MA, USA) were transfected with the two methods for 6 h and 13 h, then the samples were analyzed by confocal microscopy, fluorescence microscope and flow cytometer.

### 2.11. Quantitative Real-Time PCR Analysis

Quantitative Real-time PCR was used for the analysis of the nucleolin mRNA of HepG2 and L02. The RNA of HepG2 and L02 cells were extracted with RNAiso Plus (Takara, Osaka, Japan). The cDNA was synthesized by M-MLV Reverse Transcriptase (Vazyme Biotech, Nanjing, China). Reverse transcription PCR procedure was two-step method. The first step was 65 °C for 5 min. The second step was 42 °C for 60 min and 70 °C for 10 min. Quantitative Real-time PCR was performed by AceQ qPCR SYBR Green Master Mix (Vazyme Biotech, Nanjing, China). The primer sequences for Nucleolin for qRT-PCR are CCACTTGTCCGCTTCACACT (forward) and AGGAGCCATTTTCTTGGGGT (reverse).

### 2.12. MTT Assay

HepG2 cells were seeded on 96 well plates with 5000 cells per well, incubated with different concentrations of polybrene and treated with 10 µL MTT (3-(4,5-dimethyl-2-thiazolyl)-2,5-diphenyl-2-H-tetrazolium bromide) solution (5 mg/mL) for 4 h. After the supernatant was discarded, 100 μL DMSO (dimethyl sulfoxide) was added to dissolve the formazan. The absorbance was determined at 570 nm.

### 2.13. Lactic Dehydrogenase (LDH) Leakage Assay

HepG2 cells were seeded on 24 well plates and incubated with different concentrations of polybrene for 6 h. The supernatant was collected and centrifuged at 1000 rpm. The LDH was detected with fully automatic analyzer method (Beckman Coulter AU5800, Powhatan, VA, USA).

### 2.14. RNA Interference

The sequences of siRNA are 5-aagacagugaugaagaggagg-3 (siNCL-1#), 5-agagaucgaugggcgaucuauu-3 (siNCL-2#) and 5-uucuccgaacgugucacgutt-3 (negative control, siNC). We detected expression of NCL at 48 h.

### 2.15. Statistics Analysis

The statistical analysis was made by the IBM SPSS Statistics 19. The unpaired student’s t-test was used to compare two group. All data was showed as means ± standard deviation.

## 3. Results

### 3.1. Synthetic Procedure of Gq-DNA

**Figure 1:**
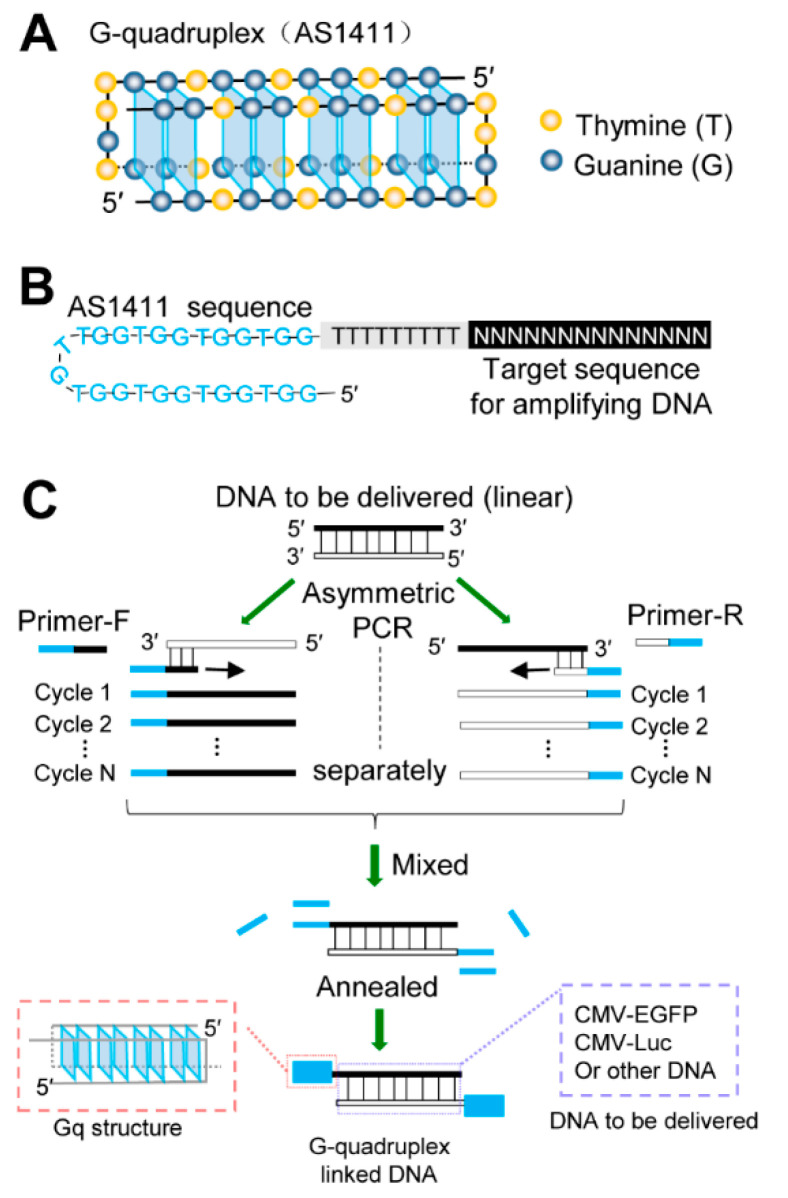
Synthetic procedure of Gq-DNA. (**A**) The structure of Gq. Every four guanines (G, blue sphere) in the four parallel DNA strands formed a guanine tetrad called G-quartet through Hoogsteen hydrogen bonding (blue parallelogram). The G-quartets were stacked into a Gq. The Gq structure was formed by two same AS1411. (**B**) Scheme of the primer designed for producing Gq-DNA. The primer contains a G-rich oligonucleotide (AS1411) to form Gq at 5′ end, which consists of a sequence for amplifying DNA at 3′ end and a poly T linker in the middle. The upstream and downstream primer sequences differ at the 3′ end according to the delivered DNA sequence. (**C**) Synthetic procedure of Gq-DNA. Firstly, a linear DNA fragment containing a promoter and a coding sequence, was acquired by conventional PCR amplification from plasmids. Secondly, the DNA single strand was linearly (non-exponentially) amplified using the linear DNA as a template with upstream or downstream primer separately, by which each single strand product containing an AS1411 sequence at 5′ end. Then, the two products amplified by upstream and downstream primers were mixed and annealed with the short free AS1411 strands. Gq-DNA was generated with complementary DNA double strands in the middle and Gq conjugated to both ends.

We introduced Gq structures, previously identified as AS1411 [20,21,22], onto the ends of linear DNA to generate Gq-DNA transfection system. Gq structure was formed by two of the same G-rich oligonucleotides derived from AS1411 (Figure 1A). Each single strand of DNA fragment with a G-rich sequence on the 5′ end was obtained by linear (non-exponential) amplification separately with primers containing AS1411 sequence (Figure 1B). The two amplified strands were annealed to form a partial double-strand DNA with cohesive ends (Figure 1C). Simultaneously, the G-rich cohesive ends folded to Gq structures together with free AS1411 (Figure 1C). In this way, we prepared Gq-linked CMV-EGFP and CMV-Luciferase (CMV-Luc) to construct two Gq-DNA transfection systems.

### 3.2. Characterization of Gq-DNA

**Figure 2:**
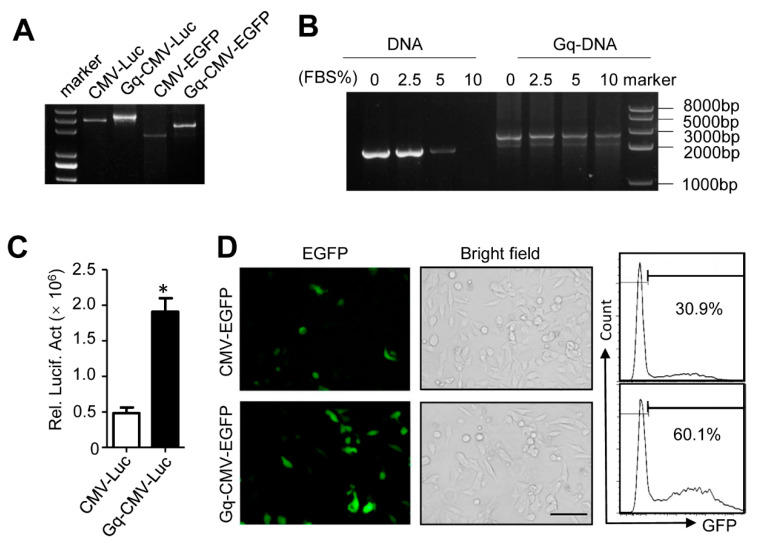
Characterization of the Gq-DNA. (**A**) The electrophoresis rate of Gq-DNA and DNA. CMV-Luc, Gq-CMV-Luc, CMV-EGFP and Gq-CMV-EGFP were analyzed by agarose gel electrophoresis. The left lane was the DNA markers of 5000, 3000, 2000, 1000, 750 and 500 bp from top to bottom. (**B**) Serum stability of Gq-DNA and DNA. Gq-CMV-EGFP and CMV-EGFP incubated with DMEM containing 0, 2.5, 5 and 10% fetal bovine serum at 37 °C for 48 h were analyzed by agarose gel electrophoresis. The rightmost lane was DNA markers of 8000, 5000, 3000, 2000 and 1000 bp from top to bottom. (**C**) Luciferase activity in HepG2 cells transfected with CMV-Luc and Gq-CMV-Luc by Lipo2000. * *p* < 0.05. Rel. Lucif. Act., relative luciferase activities. (**D**) Fluorescence microscope images (left) and quantification of flow cytometry (right) of HepG2 transfected with CMV-EGFP or Gq-CMV-EGFP by Lipo2000. Scale bar, 100 μm.

The DNA gel electrophoretic assay showed that Gq-DNA traveled more slowly than the control linear DNA (Figure 2A), probably due to the special Gq structures. DNA, especially in the linear form, is unstable in blood due to serum nuclease mediated digestion [23]. Then, we tested whether Gq could improve the stability of Gq-DNA in serum. Compared with the linear DNA that was fully degraded in DMEM containing 10% fetal bovine serum (FBS), Gq-DNA was stable in DMEM containing 10% FBS for 48 h (Figure 2B), indicating that Gq protects the linear DNA from nuclease degradation.

To determine whether Gq structure affected DNA expression, we transfected Gq-CMV-EGFP, Gq-CMV-Luciferase (Gq-CMV-Luc), and the control linear DNA into HepG2 cells using commercial transfection reagents. The activities of luciferase and EGFP fluorescence were detectable and even more effective than the control linear DNA (Figure 2C,D), demonstrating that Gq structure does not impair gene expression, and even enhanced the transfection activities, likely due to the promoted nuclear transportation.

### 3.3. Gq-DNA Transfects Tumor Cells after Neutralizing Negative Charges of Cell Membrane

**Figure 3:**
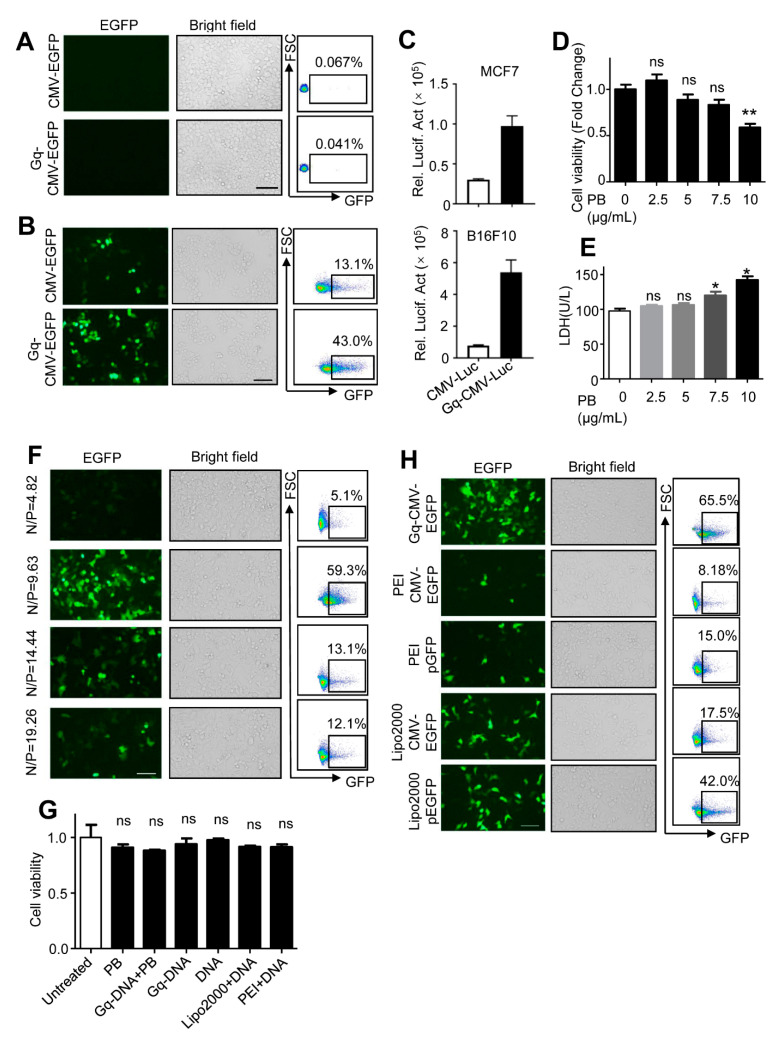
Gq-DNA transfects cells after neutralizing the negative charges of cell membrane. (**A**) Transfection efficiency of HepG2 incubated with CMV-EGFP or Gq-CMV-EGFP for 6 h. Then, 24 h later, expression of EGFP was tested with fluorescence microscopy (left) and flow cytometry (right). (**B**) Transfection efficiency of Gq-DNA transfection. HepG2 cells were incubated with polybrene (PB, 5 μg/mL) and CMV-EGFP or Gq-CMV-EGFP for 6 h. Then, 24 h later, expression of EGFP was tested with fluorescence microscopy (left) and flow cytometry (right). (**C**) Transfection efficiency of Gq-DNA transfection in MCF7 (up) and B16F10 (down) cells. Cells were incubated with polybrene (PB, 5 μg/mL) and CMV-LUC or Gq-CMV-LUC for 6 h. Then, 24 h later, expression of LUC was tested. (**D**) Cell viability of HepG2 treated with different concentrations of polybrene (PB) tested by MTT assay. NS, no significance. ** *p* < 0.01. (**E**) Permeability of cell membrane of HepG2 treated with polybrene. HepG2 cells were incubated with different concentrations of polybrene (PB) for 6 h and LDH in supernatants was tested. NS, no significance. * *p* < 0.05. (**F**) Transfection efficiency of Gq-DNA transfection at various N/P ratios. HepG2 cells were incubated with polybrene (PB, 2.5 μg/mL, 5 μg/mL, 7.5 μg/mL, 10 μg/mL) and DNA (1.6 μg) for 6 h. Scale bar, 100 μm. (**G**) Cell viability of HepG2 treated with polybrene (PB, 5 μg/mL), Gq-DNA and polybrene (PB, 5 μg/mL), Gq-DNA, DNA, Lipo2000 and DNA, PEI and DNA for 6 h and then MTT assay was carried out at 24 h. NS means no significance. (**H**) Comparison of transfection efficiency between Gq-DNA transfection, PEI and Lipo2000. HepG2 cells were treated with Gq-CMV-EGFP and polybrene (PB, 5 μg/mL) or transfected with the linear DNA (CMV-EGFP) or the plasmid expressing EGFP (pEGFP, pEGFP-C1) by PEI and Lipo2000. Scale bar, 100 μm.

Given that Gq structure could bind to nucleolin, we speculated Gq-DNA could transfect tumor cells without assistance of transfection reagents. After incubation of HepG2 cells with Gq-CMV-EGFP, HepG2 cells expressed no EGFP fluorescence (Figure 3A). We suspected that the negative charges on both cell membrane and DNA molecules prevented the binding of Gq and membrane nucleolin. To overcome this electronic rejection effect, we neutralized the negative charges with polybrene, a cationic polymer widely used to enhance viral infections similar to transfection [24,25,26,27,28,29]. As expected, the carried EGFP gene was effectively delivered into HepG2 cells and expressed after polybrene treatment (Figure 3B), indicating that Gq-DNA transfects tumor cells after neutralizing negative charges of cell membrane. The phenomenon was verified in breast cancer cell MCF7 and melanoma cell B16F10 (Figure 3C).

To assess the cytotoxicity of Gq-DNA transfection, we used MTT assay and LDH leakage assay. The concentration of polybrene we used (5 μg/mL) showed little cytotoxicity (Figure 3D,E). We tested the transfection efficiency at the various ratios of polybrene to DNA (N/P ratios). It turned out that an N/P ratio of 9.63 meant that 1.6 μg DNA and 5 μg polybrene per ml of medium produced the highest transfection efficiency (Figure 3F). Gq-DNA transfection and its components did not show more cytotoxicity than Lipo2000 or PEI (Figure 3G). In addition, Gq-DNA transfection was more efficient than PEI or Lipo2000 transfecting the linear DNA and plasmid DNA (Figure 3H and Figure S1). Taken together, Gq-DNA transfection is a low cytotoxic and effective gene delivery method.

### 3.4. Gq-DNA Transfection Effectively Targets Tumor Cells with High-Level Nucleolin

**Figure 4:**
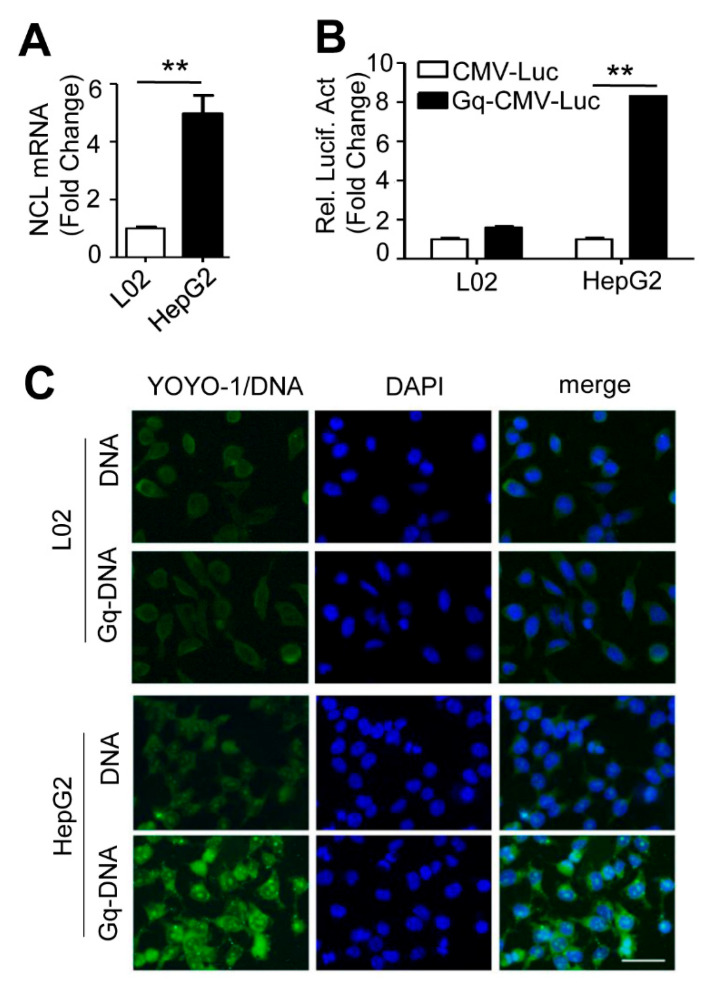
Gq-DNA transfection effectively target tumor cells with high-level nucleolin. (**A**) Relative mRNA expression of nucleolin (NCL) mRNA in HepG2 and L02. ** *p* < 0.01. (**B**) Relative luciferase activities (Rel. Lucif. Act.) in HepG2 and L02 treated with polybrene (PB, 5 μg/mL) and CMV-Luc or Gq-CMV-Luc. ** *p* < 0.01. (**C**) Cellular uptake of DNA and Gq-DNA by HepG2 cells and L02. Cells were treated with polybrene (PB, 5 μg/mL) and YOYO-1-stained DNA and Gq-DNA for 6 h, respectively. Cellular uptake efficiency was tested by fluorescence microscope. Scale bar, 50 μm.

To verify the tumor targeting capability of Gq-DNA transfection, we compared the transfection efficiencies in human hepatocellular carcinoma cell line HepG2 and normal liver cell line L02. We firstly tested the expression of nucleolin and found that nucleolin level in HepG2 cells was 3- to 4-fold higher than that in L02 cells (Figure 4A). Accordingly, Gq-DNA transfection worked more effectively in HepG2 cells, but not in L02 cells where Gq-DNA exhibited the transfection efficacy comparable to the control linear DNA (Figure 4B). The same phenomenon was verified in human colon cancer cells HCT116 and normal colonic epithelial cells NCM460 (Figure S2). Furthermore, we tracked the DNA during the process of Gq-DNA transfection by staining Gq-DNA and the control linear DNA with a fluorescent dye YOYO-1 before delivery. Microscopic images confirmed that intracellular Gq-DNA was significantly higher than the control linear DNA in HepG2 cells, but not in L02 cells (Figure 4C). These results demonstrated that Gq-DNA transfection worked more efficiently in tumor cells, indicating that Gq-DNA transfection could effectively target tumor cells with high-level nucleolin.

### 3.5. Gq-DNA Transfection Works Independently of Cell Proliferation

**Figure 5:**
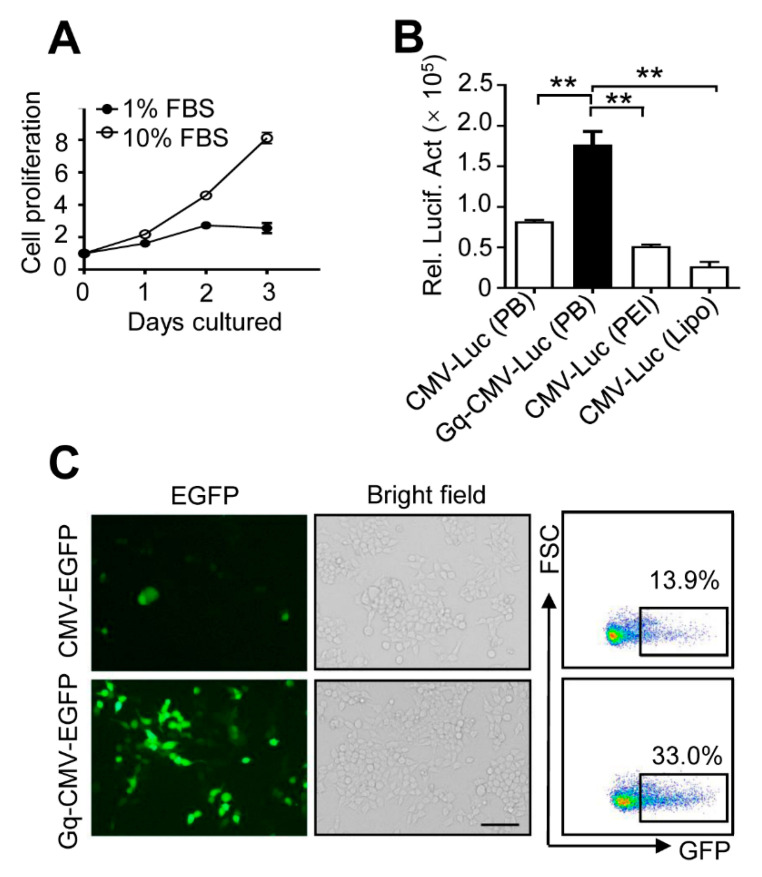
Gq-DNA transfection works independently of cell proliferation. (**A**) Cell proliferation of HepG2 treated with DMEM containing low fetal bovine serum (1%) or normal fetal bovine serum (10%) for 0, 1, 2 and 3 days. (**B**) Comparison of transfection efficiency between Gq-DNA transfection, PEI and Lipo2000 under low fetal bovine serum (1%) medium. HepG2 cells were treated with CMV-Luc and polybrene (PB, 5 μg/mL), Gq-CMV-Luc and polybrene (PB, 5 μg/mL), transfected CMV-Luc with PEI or Lipo2000. Then, 24 h later, relative luciferase activities (Rel. Lucif. Act) were quantified. ** *p* <0. 01. (**C**) Transfection efficiency of Gq-DNA transfection under low fetal bovine serum medium. HepG2 cells cultured with low fetal bovine serum (1%) medium were treated with polybrene (PB, 5 μg/mL) and CMV-EGFP or Gq-CMV-EGFP. Then, 24 h later, the expression of EGFP was tested with fluorescence microscopy (left) and flow cytometry (right). Scale bar, 100 μm.

The efficiency of conventional approaches of DNA delivery usually depends on cell proliferation and mitosis [8]. Thus, we detected the efficiency of Gq-DNA transfection when HepG2 cells were in a low rate of cell proliferation and mitosis (Figure 5A). Gq-DNA transfection could deliver luciferase gene into HepG2 cells more effectively compared with commercial transfection reagent even cultured in low serum medium (2.15-fold, 3.44-fold and 6.63-fold higher than the control linear DNA, PEI and Lipo2000, respectively) (Figure 5B). Microscopic images and flow cytometry confirmed that Gq-DNA transfection could work independently of cell proliferation and mitosis (Figure 5C).

### 3.6. Gq-DNA Transfection Depends on the Binding between G-Quadruplex and Nucleolin

**Figure 6:**
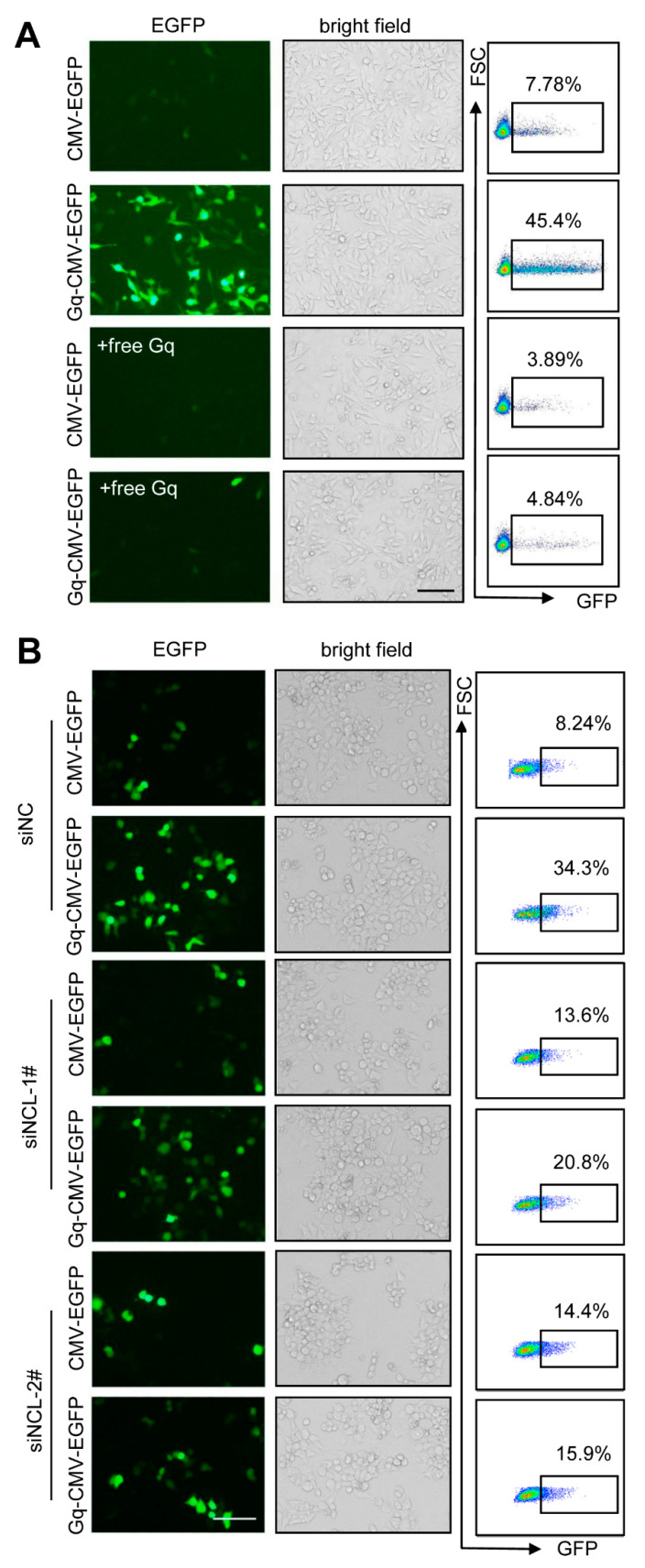
Gq-DNA transfection depends on the binding between G-quadruplex and nucleolin. (**A**) Transfection efficiency of Gq-DNA transfection after blocking the binding between nucleolin and Gq-DNA with excessive free Gq. HepG2 cells were treated with polybrene (PB, 5 μg/mL) and CMV-EGFP or Gq-CMV-EGFP and excessive free Gq (12 μg) for 6 h. Then, 24 h later, the expression of EGFP was tested with fluorescence microscopy (left) and flow cytometry (right). Scale bar, 100 μm. (**B**) Transfection efficiency of Gq-DNA transfection after knocking down the expression of NCL. HepG2 cells were transfected with siRNA targeting NCL. Then, 48 h later, HepG2 cells were treated with polybrene (PB, 5 μg/mL) and CMV-EGFP or Gq-CMV-EGFP for 6 h. Then, 24 h later, the expression of EGFP was tested with fluorescence microscopy (left) and flow cytometry (right). Scale bar, 100 μm.

To confirm the function of Gq structures in Gq-DNA transfection, we added excessive free Gq as the antagonist to block the binding of nucleolin with Gq-DNA. The results showed that excessive free Gq greatly reduced the efficiency of Gq-DNA transfection of CMV-EGFP (Figure 6A). To further verify that Gq-DNA transfection depends on the high expression of nucleolin, nucleolin expression in HepG2 cells was knock down before transfection (Figure S3). Transfection efficiency of Gq-DNA transfection decreased significantly after nucleolin knockdown (Figure 6B), indicating that Gq-DNA transfection is highly dependent on the expression of nucleolin. Altogether, Gq-DNA transfection depends on the binding between G-quadruplex and nucleolin, which further proves that Gq-DNA transfection effectively targets the tumor cells with high-level nucleolin.

### 3.7. Gq Facilitates the Entrance of Gq-DNA into Cells and Nuclei

**Figure 7:**
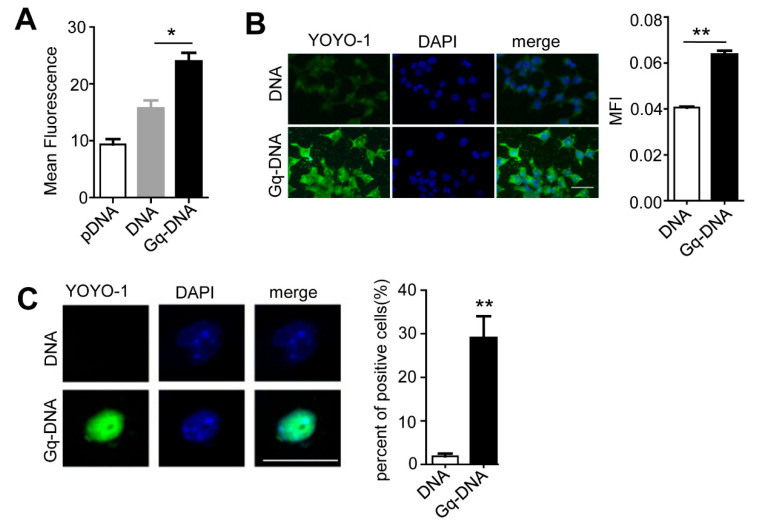
Gq facilitates the entrance of the Gq DNA into cells and nuclei. (**A**,**B**) Cellular uptake of plasmid DNA (pDNA), DNA and Gq-DNA by HepG2 cells. HepG2 cells were treated with polybrene (PB, 5 μg/mL) and YOYO-1-stained pDNA, DNA and Gq-DNA for 6 h. Cellular uptake efficiency was tested by flow cytometry (**A**) and fluorescence microscope (**B**). * *p* < 0.05. ** *p* < 0.01. Scale bar, 50 μm. (**C**) Accumulation of DNA and Gq-DNA in nuclei. HepG2 cells were treated with polybrene (PB, 5 μg/mL) and YOYO-1-stained DNA and Gq-DNA for 13 h. Fluorescent images were taken by confocal microscopy. Positive cell means cell with abundant YOYO-1 in the nuclei. Scale bar, 20 μm. ** *p* < 0.01.

As reported, Nucleolin is a shuttle protein moving between cell membrane, cytoplasm and nuclei [12]. Thus, Gq-DNA might enter tumor cells and reached nuclei along with nucleolin. We tracked the DNA during the process of Gq-DNA transfection using a fluorescent dye YOYO-1. Flow cytometry and microscopic images showed that 6 h after the delivery, intracellular Gq-DNA was significantly higher than the control linear DNA and plasmid DNA, and mainly accumulated in cytoplasm (Figure 7A,B), demonstrating that Gq promotes the entrance of Gq-DNA into HepG2 cells. Thirteen hours after the delivery, a markedly high level of Gq-DNA was found in the nuclei (Figure 7C). Therefore, Gq facilitates the entrance of Gq-DNA into cells and nuclei.

## 4. Discussion

The major drawbacks of conventional virus-mediated gene delivery are immunogenicity and the risk of randomly insertional mutagenesis. As to non-viral methods, low transfection efficiency and poor selectivity are the main bottleneck [30]. To overcome the weakness of conventional gene delivery system, we set to design a gene transfer system targeting a certain protein overexpressed in tumor cells to provide tumor targeting and deliver the nucleic acids directly into nuclei to improve transfection efficiency. To this end, we developed the low cytotoxic, efficient and tumor-targeting Gq-DNA transfection method by utilizing the interaction of nucleolin and Gq.

First, a special amplifying method of PCR was used to generate Gq-DNA that could transfect cells overexpressing nucleolin. In nucleolin-overexpressing cells, Gq-DNA bound to nucleolin on cell membrane after the negative charges of cell membrane were neutralized. Gq-DNA entered cells and reached nuclei along with nucleolin that shuttled between cell membrane and nuclei [31,32]. Then, the gene delivered by Gq-DNA expressed in the nuclei. In normal cells, Gq-DNA could not enter cells because there was almost no nucleolin expressed in cell membrane. Based on our design, Gq-DNA transfection effectively targets cells with high-level nucleolin. Nucleolin-overexpressing HepG2 cells were used as a cell model to prove this. Previous studies revealed that many kinds of tumor cells overexpressed nucleolin [33]. Thus, Gq-DNA transfection can also be used for tumor cells. We verified the effectiveness of Gq-DNA transfection for tumor cells with high level nucleolin in B16F10, HCT116 and MCF7. According to all the results, Gq-DNA transfection is low cytotoxic, efficient, targeting tumor cells with high-level nucleolin, and acts independently of mitosis.

Nuclear entry is the last intracellular barrier of exogenetic gene expression because of the nuclear envelope. Nuclear envelopes break down during the process of cell divisions. Fast dividing cells were transfected more efficiently than slow dividing cells. Conventional transfection methods only deliver nucleic acids into cytoplasm, which means that their transfection efficiency depends on mitosis. Thus, direct delivery of nucleic acids into nuclei would be an improved strategy [34]. Gq-DNA transfection can work independently of cell division and directly deliver DNA into nuclei, providing a new choice to achieve gene delivery for slow dividing cells.

More importantly, the efficiency of Gq-DNA transfection is dependent on the expression of nucleolin, which means that Gq-DNA transfection can target tumor cells with high-level nucleolin. Nucleolin, besides high expression on the tumor cell surface, was also found to express on the surface of endothelial cells during angiogenesis [35]. To support rapid proliferation, tumor cells secrete pro-angiogenic factors to promote angiogenesis [36]. Thus, nucleolin can be a target for potential therapy against both tumor cells and tumor blood vessel. Due to lack of selectivity for tumor, intratumoral injection is the most common method of current tumor gene therapies. Whereas it limits the application of gene therapies in some tumors that are difficult to intratumorally inject [2]. The stability of Gq-DNA in serum makes systemic administration possible. Gq-DNA transfection provides a potential way for cancer selective DNA delivery. Although it was reported that polybrene (10 micro g/muscle) was injected in muscles to improve lentivirus delivery [37]. At the moment, Gq-DNA transfection can only be used as a transfection method in vitro due to the lack of enough studies using relevant preclinical animal models. The application of Gq-DNA transfection in vivo needs further research, which is our next step.

Unlike the traditional chemical transfection methods, Gq-DNA entered cells and nucleus by ligand dependent uptake mechanisms after neutralizing negative charges of cell membrane. The advantages of Gq-DNA transfection are high efficiency (comparing with PEI and Lipo2000) and low cytotoxicity. Correspondingly, the disadvantages of Gq-DNA transfection are that Gq-DNA is in an unfolded loose state which will increase the steric hindrance and decrease the diffusion rate. The longer the strand of dsDNA, the greater the impact on steric hindrance and diffusion rate. Thus, there would be a maximum size for delivery. However, we cannot confirm exact value of the maximum size.

Additionally, Gq-DNA is delivered directly into nuclei which depends on the shuttle of nucleolin between cell membrane, cytoplasm and nuclei. Thus, we speculated that the genes that might affect shuttle of nucleolin were inappropriate to Gq-DNA transfection. Similar to traditional chemical transfection methods, Gq-DNA transfection is transient transfection and not superior in durability.

Indeed, the expression of nucleolin is significantly enhanced in several solid tumors such as pancreatic cancer, hepatocellular carcinoma, colorectal cancer, thyroid cancer, glioma, breast cancer, prostate cancer, gastric cancer, melanoma and leukaemia [30]. Gq-DNA transfection can be applied in these nucleolin-overexpressing tumors. However, as to normal cells, transfection efficiency of Gq-DNA transfection is low which restricts its application as a transfection method in vitro.

## 5. Conclusions

In this study, Gq-DNA transfection was designed as a vector-free gene transfer strategy for selective delivery of DNA to cancer cells. Our work demonstrates that: (i) Gq-DNA transfection is a low cytotoxic and efficient strategy for gene delivery; (ii) Gq-DNA transfection selectively deliver DNA into tumor cells with high-level nucleolin; (iii) Gq-DNA transfection work independently of cell division by directly delivering DNA into nuclei; (iv) Gq-DNA transfection is characterized by ease of synthesis and low cost (Cost Accounting is in the Supplementary Materials).

## Supplementary Materials

The following supporting information can be downloaded at: https://www.mdpi.com/article/10.3390/pharmaceutics14102247/s1. Figure S1: Comparison of transfection efficiency between Gq-DNA auto-transfection and Lipo2000; Figure S2: Relative luciferase activities in NCM460 and HCT116; Figure S3: Nucleolin expression of HepG2 transfected with siNCL; Cost Accounting.
