# Supplementary material for: G-Quadruplex Linked DNA Guides Selective Transfection into Nucleolin-Overexpressing Cancer Cells
Source: Pharmaceutics. 2022 Oct 21;14(10):2247. doi: 10.3390/pharmaceutics14102247 (PMC9609445; doi:10.3390/pharmaceutics14102247)
Supplement: Supplementary file 1 [file pharmaceutics-14-02247-s001.zip › pharmaceutics-1838194-supplementary.pdf]

Supplementary Material

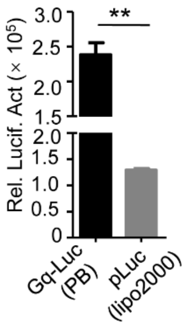

Figure S1. Comparison of transfection efficiency between Gq-DNA auto-transfection and Lipo2000. HepG2 cells were treated with Gq-CMV-Luc and polybrene (PB, 5 μg/mL) or transfected with the plasmid expressing luciferase (pLuc, pCMV-tag2B-luciferase) by Lipo2000. 24 hours later, relative luciferase activities (Rel. Lucif. Act) were quantified. **, $P < 0.01$.

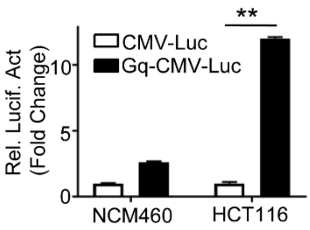

Figure S2. Relative luciferase activities (Rel. Lucif. Act.) in NCM460 and HCT116 treated with polybrene (PB, 5 μg/mL) and CMV-Luc or Gq-CMV-Luc. **, $P < 0.01$.

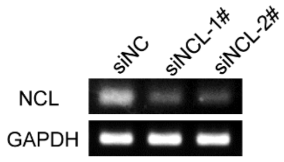

Figure S3. HepG2 cells were transfected with siNC (scrambled siRNA serves as negative control), siNCL-1#, and siNCL-2#. 48 hours later, the expression of NCL were detected by Semi-quantitative PCR.

Cost Accounting.

Critical reagents needed to produce Gq-DNA are DNA Polymerase (2 × Rapid Taq Master Mix, Vazyme Biotech, Nanjing, China, $2.4 per mL) for PCR reaction and PCR Product Purification kit (SIMGEN, Hangzhou, China, $42/50 reactions) to remove free Gq. These reagents that cost about $20 can produce sufficient Gq-DNA (about 150 μg) because of high efficiency of PCR

reaction. Thus, compared with conventional transfection regent, for example, 0.75 mL Lipofectamine™ 2000 that transfects about 300 μg DNA sells for 479 dollars, Gq-DNA transfection is low cost.
